# Understanding tile drain discharge dynamics through saturated area observation

**DOI:** 10.1038/s41598-026-56451-3

**Published:** 2026-06-11

**Authors:** Dušan Marjanović, Juraj Parajka, Borbala Szeles, Camillo Ressl, Peter Strauß, Elmar Schmaltz, Günter Blöschl

**Affiliations:** 1 Institute of Hydraulic Engineering and Water Resources Management, TU Wien, 1040 Vienna, Austria; 2 Department of Geodesy and Geoinformation, TU Wien, 1040 Vienna, Austria; 3 Institute for Land and Water Management Research, Federal Agency for Water Management, 3252 Petzenkirchen, Austria

**Keywords:** Tile drainage, Saturated area, Hydrological connectivity, Time-lapse photography, Environmental sciences, Hydrology, Natural hazards

## Abstract

This study explored the potential of using saturated area patterns in understanding tile drain response from an agricultural hillslope. Using spatial analysis of time-lapse imagery gathered from the Hydrological Open-Air Laboratory (HOAL) in Petzenkirchen, Lower Austria, twelve events between 2015 and 2017 have been analyzed. Visual inspection of the analyzed events reveals connected or disconnected saturation patterns in the downslope direction of the hillslope. The connected events show consistently larger average maximum connected distance (> 32 m) and average saturated area (> 50 m^2^) during the events. The results show that for the connected events, tile drain response is always low, while for the events that become connected, the tile drain response increases rapidly with connectivity distance. Overall, the observed saturated areas are shown to outperform traditional explanatory variables of tile drain response, with the exception of event groundwater levels, with a 59% and 62% of behaviour explained for peak tile drain discharge and event volume, respectively. Camera observations are sensitive to snow cover and surface vegetation, which necessitates a manual pre-screening of the captured events for possible use. Overall, the findings of this study suggest that observations of saturated areas are highly informative for predicting hillslope scale runoff and related hydrological processes because they provide spatially distributed rather than point scale information, which presents a cost-effective method with potential of much wider use in hydrology.

**Supplementary Information:**

The online version contains supplementary material available at 10.1038/s41598-026-56451-3.

## Introduction

Artificial drains are a common practice to improve soil moisture and aeration conditions on agricultural lands with naturally high groundwater tables^1^. The removal of excess water through tile drainage leads to an accelerated drying of the soil at the beginning of the growing season and lengthens the growing season. The widespread usage of tile drainage, coupled with the fact that they significantly contribute to the transport of nutrients and pesticides to surface water, which in turn causes contamination and eutrophication, requires a better understanding of the controls and processes that govern the discharge behaviour of tile drains^2,3^.

The hydrologic response of tile drains is controlled by rainfall characteristics, soil saturation conditions, and the dynamics of the shallow groundwater table^4^. Previous studies have observed the episodic nature of tile drain runoff responses to rainfall and snowmelt events^5–10^, as well as the dynamic response of the tile drainage systems to precipitation events, with rainfall intensity and antecedent moisture conditions determining the speed and magnitude of subsurface discharge^11,12^. Tile drains impact the timing, shape, and water yield of surface runoff hydrographs by affecting the underlying processes following precipitation^13^, with the tile drainage system increasing peak discharges^14^. Rainfall driven tile drain response results from complex interactions among water infiltration, soil saturation, and a shallow groundwater table; where changes in precipitation can rapidly activate or deactivate drainage pathways. Tile drain response is highly dependent on how soils become saturated and when saturation occurs^8,15–18^. A study conducted in the Hydrological Open-Air Laboratory (HOAL) found that tile drainage dynamics were strongly linearly correlated with soil moisture and also exhibited a pronounced non-linear relationship with it^10^. As shown in, e.g.,^19^ the timing of tile drain activation varies significantly with soil properties and seasonal conditions. The degree of soil saturation directly controls which flow pathways become active during precipitation events and how much precipitation can bypass the soil matrix through macropores^20^. When saturated conditions develop, preferential flow through macropores can become a dominant transport pathway^21^. Another factor controlling tile drain response is the connectivity of saturated areas. Connectivity of saturated areas refers to the degree to which water-saturated zones are hydraulically linked through subsurface pathways and to stream networks, enabling water transmission and flow generation^22^. The state of saturation and connectivity of a saturated area is thus an important factor controlling the tile drain response.

There are various methods for observing saturated areas and their connectivity, ranging from traditional field techniques to advanced instrumental and remote sensing approaches^23–25^. The level of soil saturation can be inferred from conventional soil moisture measurements using soil moisture tensiometers, capacity or resistivity sensors^26,27^ or direct measurements of shallow groundwater fluctuations using observation wells and piezometers^28^, which allow tracking changes in saturated zone depth over time in response to precipitation and evapotranspiration. Recent innovations and developments in Internet of Things platforms have enabled the application of low-cost automated soil moisture monitoring systems, which integrate multiple measurements of soil moisture to assess spatial patterns of saturation^29^. The use of soil moisture monitoring systems on agricultural fields with regular land management practices is, however, limited.

An alternative way for its estimation is the use of remote sensing methods. This includes the use of microwave or multispectral satellite imagery^30–32^ or thermal and optical cameras^33,34^. Passive microwave radiometry, based on spectral measurements in the millimetre to decimetre wavelength range, allows detection of saturated areas and water seepage through irrigation constructions^30^. On the other hand, multispectral satellite imagery from platforms such as Landsat or Sentinel provides an indirect way that allows for the derivation of vegetation and water-related indices that correlate with soil saturation^31^. The Normalized Difference Water Index can be used to indicate changes in crop water content, thereby assessing critical root zone dynamics and saturation conditions^31^. The spatial resolution of satellite imagery (i.e., 10–20 m) is, however, more suitable for larger catchment scales than for detailed investigation in agricultural fields. The high spatial resolution sampling of saturated areas (in centimeter-scale details) is possible with portable thermal infrared cameras during field visits^33,35^. Saturated soils have higher thermal inertia and lower surface temperatures compared to unsaturated areas, due to evaporative cooling, which creates a clear visual and quantitative distinction that can be used for mapping^33^. This method works best under specific conditions, i.e. cloud-free skies, suitable solar illumination for creating thermal contrasts, and limited vegetation coverage. Still, it requires field visits, as the sensing distance is relatively short and cannot be used for the mapping of larger areas. An alternative is the use of optical digital cameras, which allow for automatic mapping of saturated areas in a high temporal resolution (i.e., minutes) and spatial resolution (at the centimeter scale) over an agricultural field^34^. The previous studies, however, did not explore the use of such a mapping approach for evaluating the role of saturated areas on tile drain response.

While previous research indicates that peak flow responses to tile drain discharge response depend on event intensity and antecedent conditions^36^, there is limited knowledge about the impact of the spatial and temporal evolution of saturated areas on tile drain peak discharge magnitudes. Previous studies have identified the roles of rainfall^16,37–40^, antecedent soil moisture^38, 41^, and shallow groundwater conditions^41^; however, the patterns and connectivity of saturated zones that control tile drain response during individual storm events remain poorly understood.

The main objective of this study is to investigate the value of digital camera observations of saturated areas in estimating the tile drain response, in terms of peak tile drain discharge and total drained volume, in a small agricultural basin. Specifically, the study aims to: (i) analyse the potential of time-lapse photography to estimate the connectivity and temporal evolution of saturated area patterns; (ii) evaluate the relationship between saturated area and factors controlling tile drain response.

## Study area and data

### Study area

**Fig. 1:**
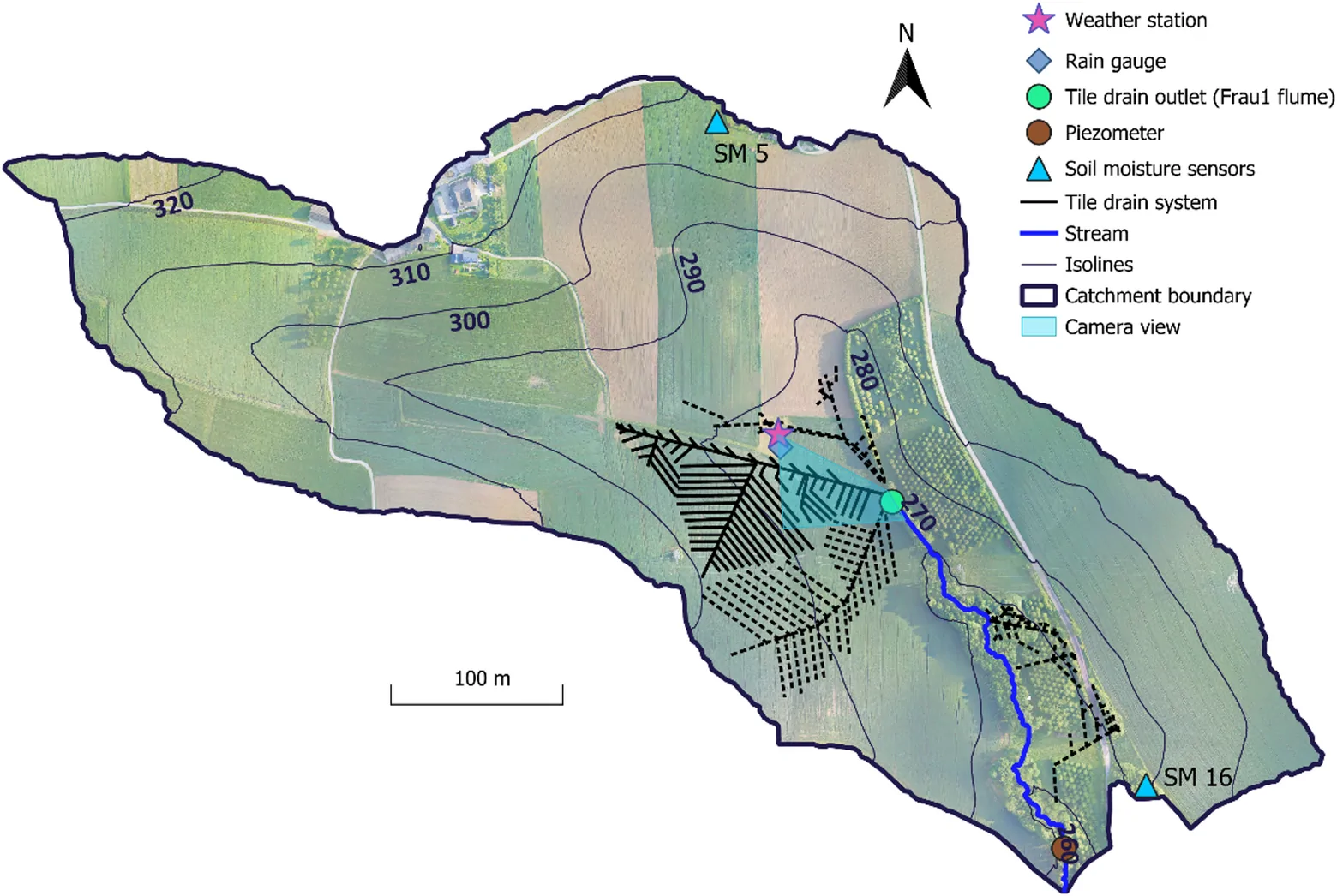
The orthophoto of the Hydrological Open Air Laboratory (HOAL), Petzenkirchen, Lower Austria. Black lines show the location of the analyzed tile drain system, with the solid line indicating the part of the system draining into the Frau1 flume, and symbols indicate the locations of stations observing hydrological and meteorological data. The camera view is shown with the cyan area.

The study is conducted at the HOAL, an experimental catchment (Fig. 1) located in Petzenkirchen, Lower Austria. The catchment area at the outlet is 66 ha. The elevation of the catchment ranges from 268 to 323 m a.s.l. with a mean slope of 8%. The HOAL is situated in a temperature humid climate with a mean annual precipitation of 744 mm yr^− 1^ (2015–2017). It is special in that various runoff generation mechanisms can be observed simultaneously across different parts of the catchment^42^.

At present, 87% of the catchment area is arable land, 5% is pasture, 6% is forested and 2% is paved. The crops are mainly winter wheat and maize. The soils are generally classified as silt loam^43^. Due to shallow, low permeability soils and the use of the catchment area as agricultural land, the concave part of the catchment was tile-drained in the 1940s to reduce waterlogging. The estimated drainage area from the tile drains is about 15% of the total catchment and can be divided into two larger systems in the south-western part of the catchment and four smaller drainage systems in the north-eastern part^42^. The tile drain system analysed in the study drains into the main stream at the Frau1 site (Fig. 1).

### Camera observations

To capture the saturated areas, images from a 2-megapixel optical camera (Sanyo VCCMCH5600P) installed on the weather station mast (purple star on Fig. 1) are used. The camera view (cyan area on Fig. 1) focuses on a 0.3 ha area with approximate dimensions of 105.3 × 25.9 m. Time-lapse pictures are taken every minute between civil dawn and civil dusk (i.e., approximately 30 min before sunrise and after sunset). The dataset of camera observations used in this study spans from January 2015 to December 2017.

**Fig. 2:**
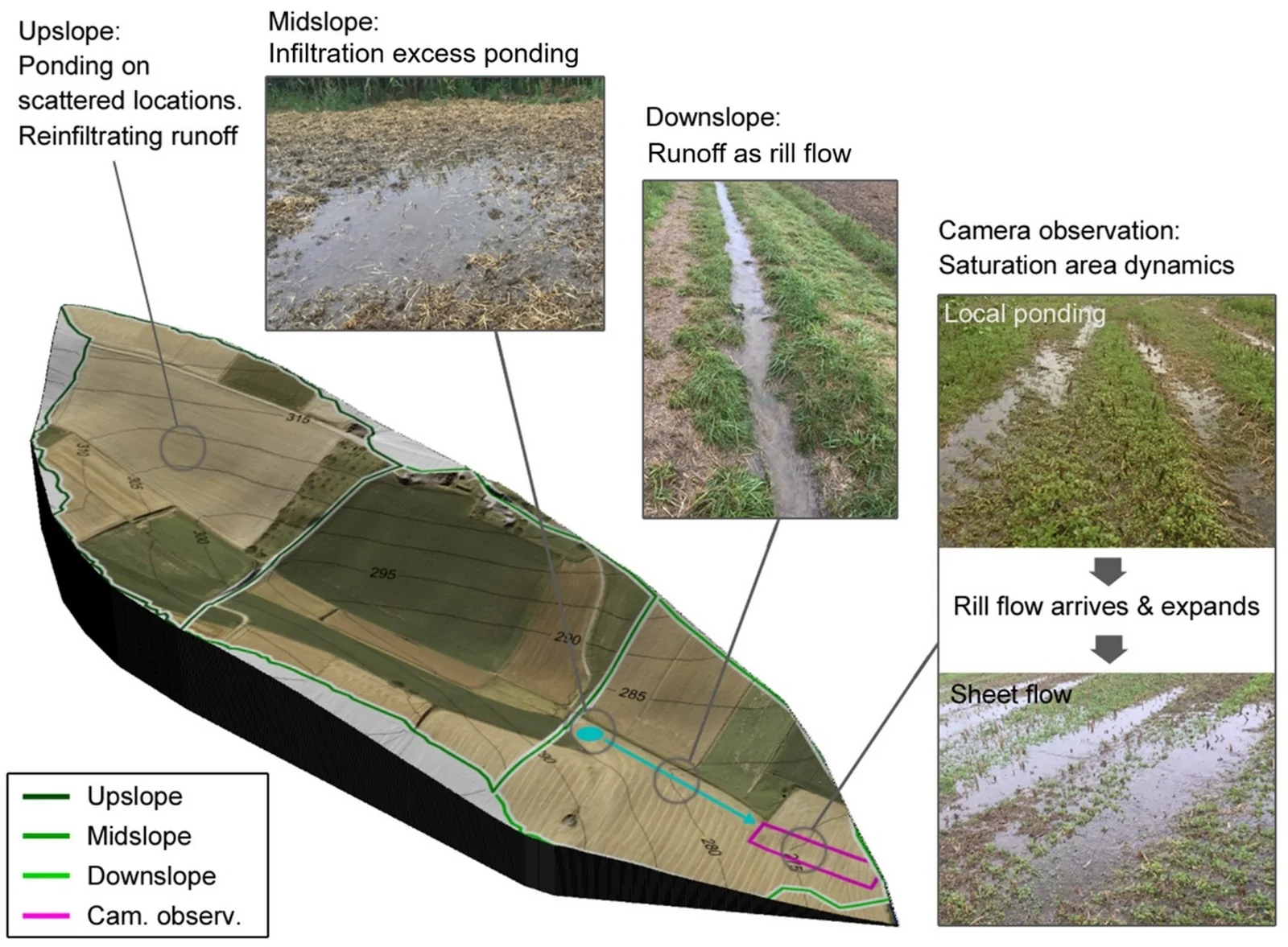
Three parts of the hillslope contributing to the tile drain runoff, and photos of runoff generation processes, during the rainfall events.

The observed saturation areas occur in a depression zone (thalweg) of an agricultural field in the downslope part of a 26.8 ha hillslope (Fig. 2). Based on the average slope (S) and the field border, the hillslope consists of three parts: downslope (S = 3%), midslope (S = 1%), and upslope (S = 7%). The soils in this area consist of cambisols (upslope part), colluvisols (middle part), and gleysol (downslope part). A hardpan horizon at 30 cm depth is due to soil cultivation practices and is also found in other similar sites. Figure 2 shows the runoff generation processes along the three sections typically observed during rainfall events. At the upslope area, saturation area occurs as scattered small ponding spots and occasional runoff, which infiltrates as it flows downstream. No surface run-on from the upslope area is connected to the midslope area. At the midslope, due to the converging topography, rainfall excess forms a pond at the foothill area where it transitions to the downslope area. When this midslope pond spills excess runoff, it flows downstream as rill flow along the upper part of the downslope. When the run-on reaches the lower part of the downslope, where the camera observation site is located, local ponding usually already occurs in this area. As the run-on enters the ponding area, the flatter topography reforms the rill flow into sheet flow.

### Tile drain runoff events

Tile drain runoff is measured at Frau1, where a precalibrated H-type flume monitors runoff at a 1-min resolution. Rainfall data associated with the tile drain runoff is collected at a 1-min resolution using a rain gauge (OTT Pluvio) located at the weather station (Fig. 1). For the attribution of the role of shallow groundwater, groundwater levels are observed at a piezometer located at the downstream section of the stream (brown circle on Fig. 1).

**Fig. 3:**
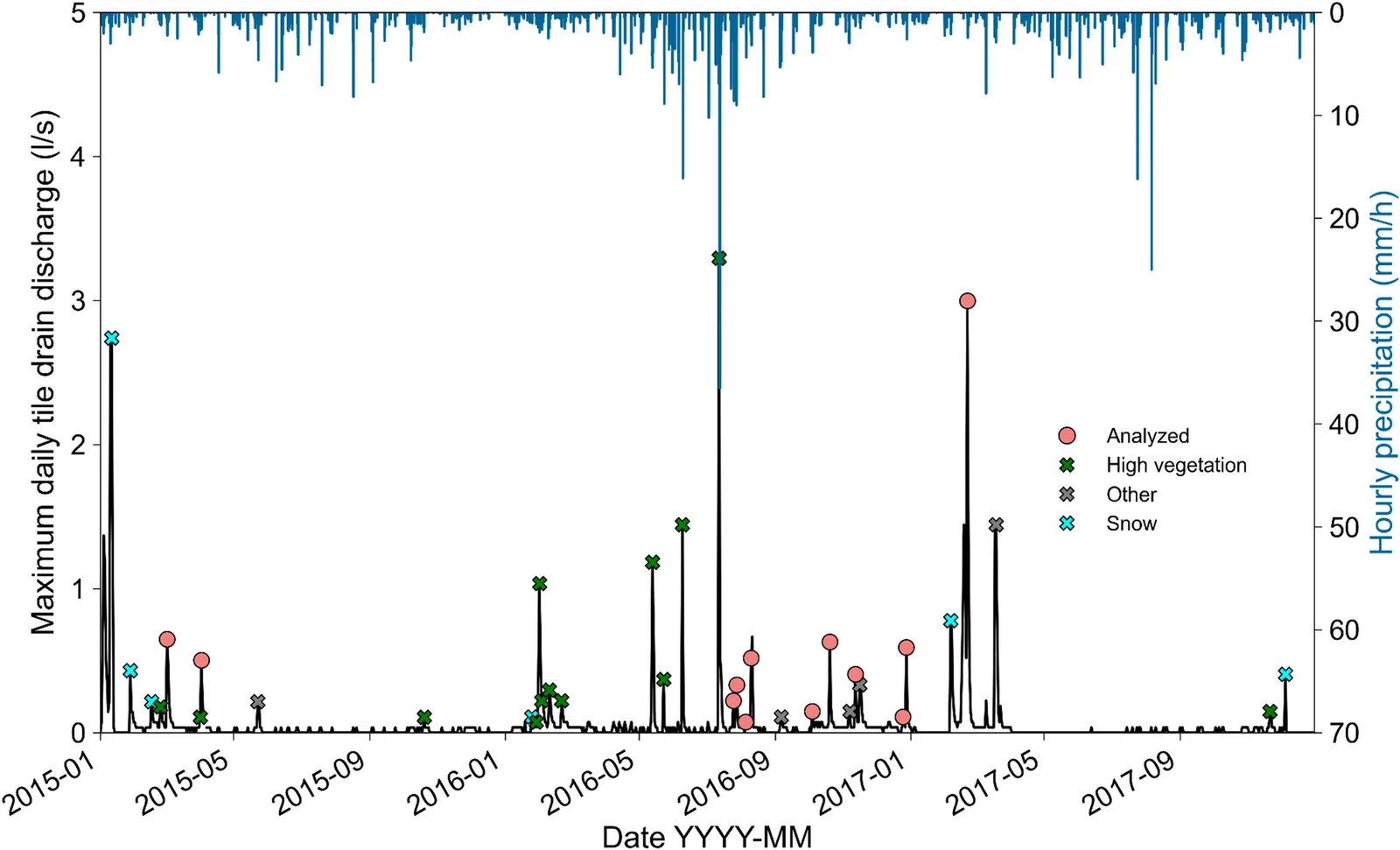
Identified tile drain runoff events during the period 2015–2017. Symbols indicate tile drain runoff peaks. Circles and x-symbols show events with available and missing camera observations, respectively. Maximum daily tile drain discharge represents the maximum daily peak of tile drain runoff.

The flow at Frau1 is ephemeral, occurring only during rainfall events. Figure 3 shows identified tile drain runoff events and availability of camera observations in the period of January 2015 to December 2017. In total, 36 runoff events are identified, however, only for 12 can the saturated area dynamics be identified from digital camera images. Most of the excluded events are due to observed snow cover (6 events) and to crops being too tall and obstructing the view (13 events). A smaller number of events is excluded due to runoff peaks occurring during the nighttime (2 events), missing camera images (2 events), and a case of no saturated areas occurring on the surface (1 event). The hydrological characteristics associated with the tile drain runoff events are described in Table 1, and a list of identified events with available camera observations is presented in Table 2.

**Table 1:** Descriptions of the selected hydrological characteristics used for analyzing tile drain response.

Hydrological characteristic	Description	Unit
Q_*p*_	Peak tile drain discharge during an event	l/s
V	Total volume drained during the event	m^3^
A_*s*_	Saturated area corresponding to the peak of the tile drain discharge	m^2^
I	Connectivity scale index corresponding to the peak of the tile drain discharge	m
hmax	Maximum connected distance between two pixels corresponding to A_*s*_	m
P_*p*_	Cumulative rainfall from the start of the event until the peak of the tile drain discharge	mm
P_I_	Average rainfall intensity from the start of the event until the peak of the tile drain discharge	mm/h
P_a_	30 day antecedent rain, serves as an indicator of initial soil wetness	mm
W_*i*_	Initial groundwater level	m
W_*max*_	Maximum groundwater level reached during the event	m
VARI	The mean of the positive values of the Visible Atmospheric Resistance Index (Gitelson et al.^44^)	–

**Table 2:** List of identified tile drain runoff events with available camera observations and associated hydrological characteristics.

Event ID	Date	Q_*P*_	V	*P*_*P*_	*P*_I_	*P*_A_	W_i_,	W_max_	VARI
1	02/03/2015	0.65	63.9	11.9	0.67	25.07	258.3	258.35	0.05
2	02/04/2015	0.5	25.1	6.34	0.26	44.16	258.32	258.35	0.67
3	25/07/2016	0.22	2.9	8.88	11.75	142	258.08	258.25	0
4	28/07/2016	0.33	13.7	17.52	3.5	122.35	258.16	258.26	0.02
5	05/08/2016	0.07	0.8	14.25	3.81	93.03	258.13	258.22	0.03
6	10/08/2016	0.52	19	18.3	0.9	85.34	258.19	258.28	0.34
7	04/10/2016	0.14	7.7	10.15	3.38	85.05	258.26	258.3	0.04
8	20/10/2016	0.63	33.8	9.08	0.6	69.81	258.32	258.36	0.16
9	12/11/2016	0.41	30.2	10.18	0.94	56.22	258.31	258.36	0
10	25/12/2016	0.09	6	5.1	0	22.37	258.26	258.29	0.02
11	28/12/2016	0.59	23.8	11.95	0.23	26.81	258.28	258.33	0.03
12	21/02/2017	1.44	164.3	8.47	0.36	28.96	258.32	258.34	0

Description of the hydrological characteristics is given in Table 1.

## Methods

**Fig. 4:**
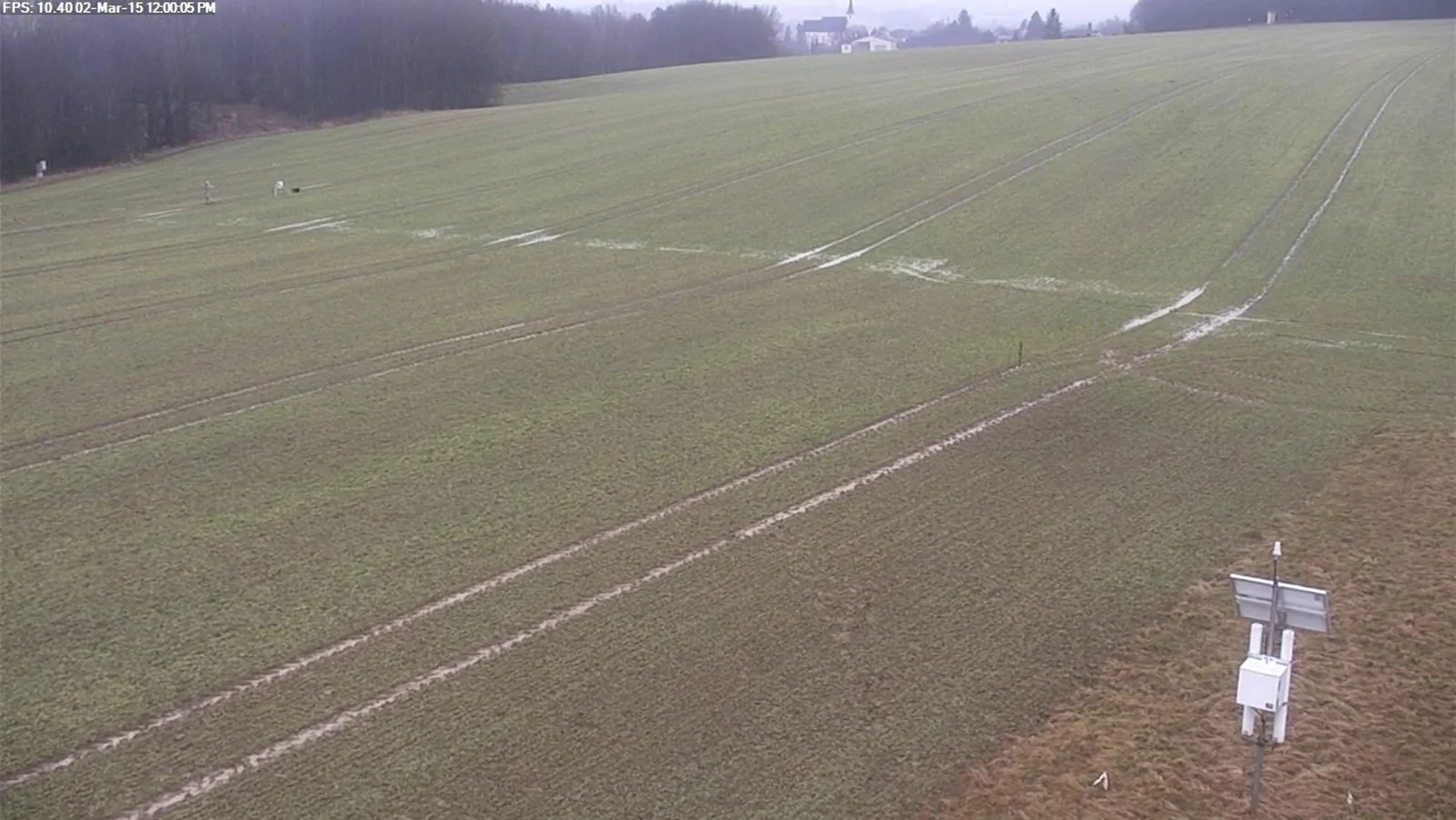
Camera view of the observed HOAL hillslope. Original image, 12:00 on March 2, 2015.

**Fig. 5:**
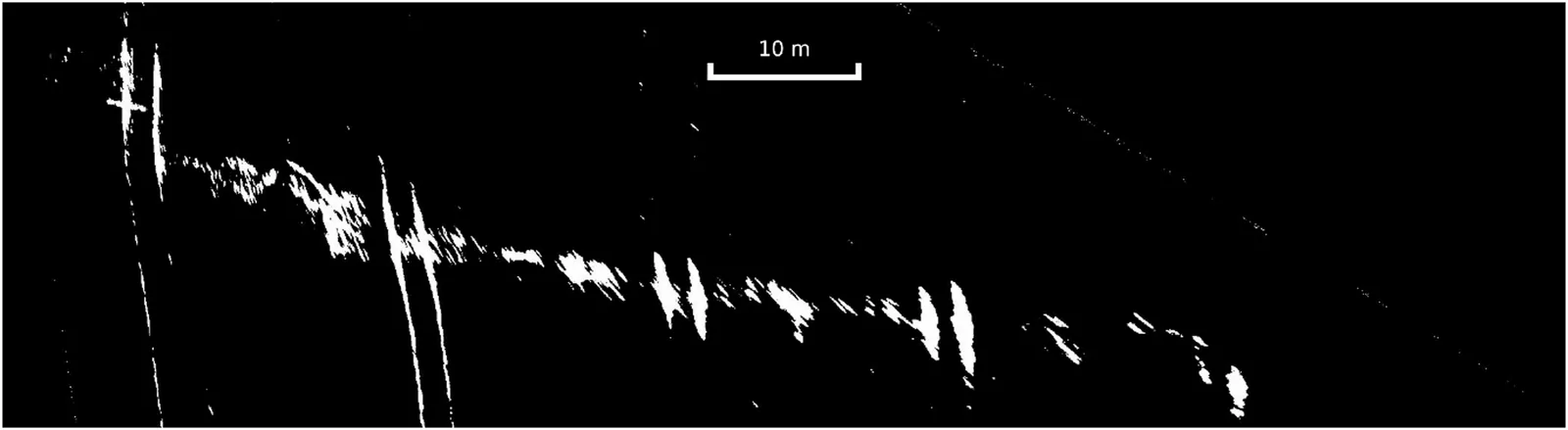
Detected saturated areas on the observed HOAL hillslope for the original image (Fig. 4) at 12:00 on March 2, 2015. Image resolution is 5 × 5 cm.

The method for obtaining saturated areas using time-lapse photography is based on a stepwise procedure described by^34^. The camera pictures (1200 × 720 pixels) are in the first step georectified to obtain a 5 cm resolution ortho-image. An example of a camera image obtained on March 2, 2015 is shown on Fig. 4. In the second step, the images are classified into binary pixels of saturation area and non-saturation area (Fig. 5) based on the greyscale histogram mode with a fixed threshold C (C = 19, based on^34^). The classification threshold was determined through a sensitivity analysis performed by^34^, which showed that the fixed threshold with a variable mode showed second-best results, second only to a variable threshold C for every event. Positional verifications against field measurements in^34^ document a good agreement with field measurements (with an RMSE = 23 cm). The final images provide spatial patterns of saturation area with a spatial and temporal resolution of 5 cm and 1 min, respectively.

### Integral connectivity scale

The connectivity of saturated areas is estimated using the approach developed by Western et al.^45^. The connectivity of the saturated area is represented as the lag-dependent connectivity function $\tau \left(h\right)$, which is estimated as the probability of a pixel at location *x* being connected to a pixel at a distance h from x, as described in Eq. 1:

1 $$\tau \left( h \right)=P\left( {x \leftrightarrow x+h{\mathrm{|}}x \in A,~x+h \in G} \right)$$

where *A* is a cluster of connected pixels in which a saturated area was detected, and *G* is the domain. The connectivity scale $I\left(m\right)$ is calculated as the integral of the connectivity function, as described in Eq. 2:

2 $$I=~\int\limits_{0}^{{{h_{max}}}} {\tau \left( h \right)dh}$$

where ${h}_{max}\left(m\right)$ is the maximum distance between two connected pixels.

The integral connectivity scale represents the average connected distance of all pixels in the domain, thus representing the degree of connectivity. For random patterns with a patchy structure, *I* is small, while for structured patterns with a high spatial connectivity, *I* is large. The mean values of these parameters during the whole event are used in the analysis for event classification.

### Analysis of factors controlling tile drain runoff response

The relationship between saturated area and connectivity indices, as well as the associated tile drain response, is analyzed in two steps: In the first step, camera images are used to classify the events into two groups: connected and disconnected events. Connected events are those where the saturated area patterns are disconnected along the entire downhill section of the hillslope. The classification is based on visual inspection of the camera images.

In the second step, the relationship between the maximum saturated area during the runoff events and the tile drain response (i.e., peak tile drain runoff and event volume) is evaluated for different hydrological conditions of the events. The hydrological characteristics are described in Table 1. The event volume is estimated using the approach of^46^, which integrates runoff volumes from the start of the event to a period of 48 h after the rainfall, or until a new rainfall episode exceeding 1 mm occurs. A statistical power analysis was conducted for an independent-samples t-test at a significance level of α = 0.05. The standardized effect size was quantified using Cohen’s d, calculated from the observed difference between the group means divided by the pooled standard deviation^47^. The observed effect size was subsequently used to estimate the achieved statistical power (1−β), representing the probability of correctly rejecting the null hypothesis when a true effect exists. This procedure follows established approaches for power estimation in two-sample comparisons^47,48^. Pairwise relationships between the two tile drain response metrics and the eight explanatory variables were quantified using both Pearson and Spearman rank correlation coefficients. 95% confidence intervals for each correlation coefficient were obtained using the Fisher z transform^49^, with the standard error computed as 1/√(*n* − 3) for Pearson and √(1.06/(*n* − 3)) for Spearman, following the adjustment proposed by^50^. Given that 16 pairwise tests were performed per correlation method (8 explanatory variables × 2 response variables), p-values were adjusted using the Benjamini–Hochberg procedure^51^ to control the false discovery rate (FDR) at 0.05, applied within each correlation method.

## Results

### Connectivity and saturation area dynamics from digital camera images

**Fig. 6:**
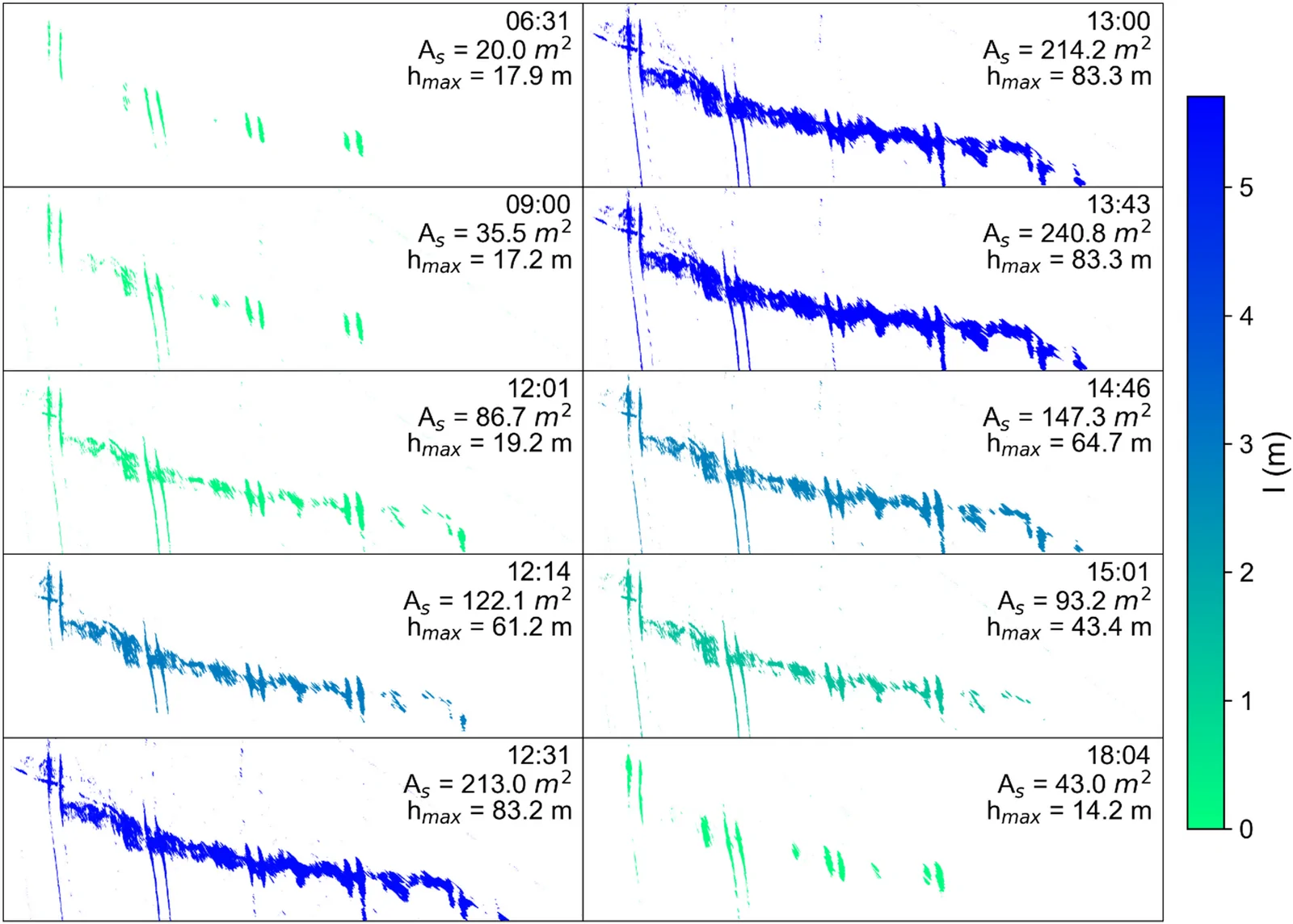
Saturation area spatial patterns at different points in time during the event on the 2nd of March 2015. The upper right corner of the subplots, and their colour, shows the timing and characteristics of the patterns: ${A}_{s}$ is the detected saturated area, ${h}_{max}$ is the maximum patch distance, and *I* is the connectivity scale value.

**Fig. 7:**
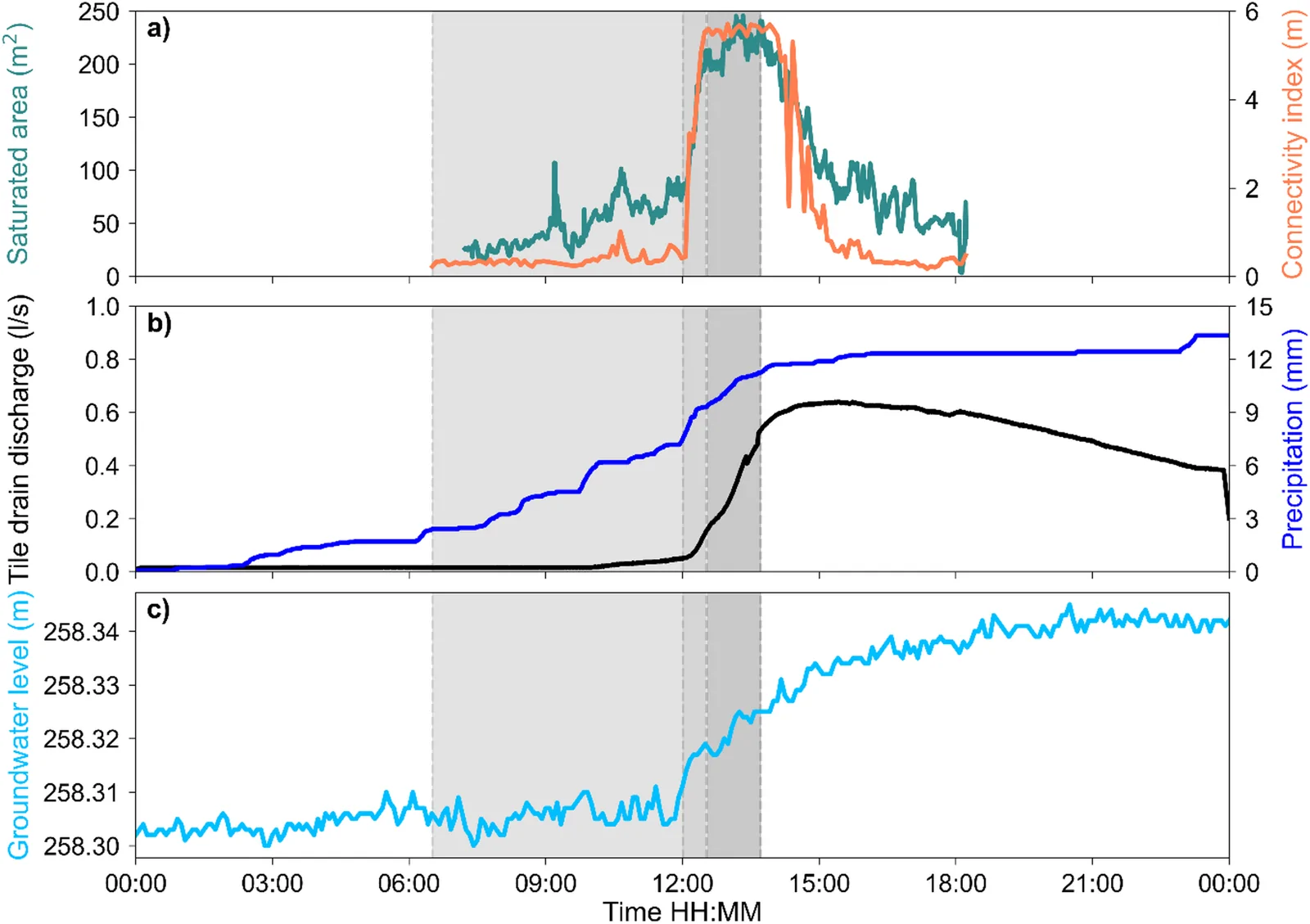
Event dynamics showing the response of the physical characteristics of the system: (**a**) The detected saturated area (teal) and the connectivity index (coral). (**b**) The precipitation (blue) and the tile drain discharge as a 15-min moving average window (black). (**c**) Groundwater level (light blue) change over the event. The vertical grey lines represent the different phases of the plots on Fig. 6.

The temporal evolution of saturation area dynamics and its connectivity is, for one example event, presented in Figs. 6 and 7. The event consists of three dynamical phases identified in^34^.

The first phase begins from 06:31 to 12:00, during which disconnected small ponds form, and the saturated area and connectivity increase from 20 to 86.7 m^2^ and 0.23 to 0.41 m, respectively. The associated maximum distance between connected pixels exceeds 19 m at the end of this phase. During this phase groundwater levels remain stable (Fig. 7).

The second phase begins at 12:01 and lasts until 12:31. During this phase, the saturated area and maximum distance between connected points increase significantly, from 86.7 to 213 m^2^ and 19.2 to 83.2 m, respectively. The tile drain system starts to activate, and connectivity increases from 0.41 to 5.53 m. Enough water ponds on the surface to overcome the microtopographical differences for the patches to connect, as indicated by the almost 13.5-fold increase in the connectivity index value at the end of the phase. In this phase, precipitation intensity and groundwater levels increase. The groundwater level precedes the activation of the tile drain by approximately 30 min. In contrast, the formation of the saturated area precedes tile drain activation by approximately 15 min.

The third phase begins at 12:32 and lasts until 13:43, after which the saturated area starts to decrease. During this phase, most individual ponds in the central part of the hillslope are already connected, and runoff occurs continuously, resulting in more moderate changes in saturation and connectivity than in the previous phase. The saturated area increases from 213 to 240 m^2^, while the maximum distance between connected points remains nearly constant (83.2 m to 83.3 m). Connectivity increases slightly from 5.53 to 5.65 m (Fig. 6). Rainfall intensity remains comparable to earlier phases, tile drain discharge approaches its peak, and groundwater levels continue to rise (Fig. 7).

Following this phase, rainfall intensity decreases and saturated areas contract and disappear, initially through runoff and subsequently through infiltration as runoff ceases. This period is marked by a steady decrease in saturated area and a sharp decline in connectivity, after which connectivity stabilizes until the end of the observation period. Tile drain discharge reaches its peak shortly after the start of saturated area contraction and then gradually decreases in the absence of sufficient rainfall, while groundwater levels flatten toward the end of the day (Fig. 7).

Consistent with saturation-excess runoff generation, hillslope responses follow a clear temporal sequence dominated by subsurface processes. Groundwater levels initially remain stable but begin to rise prior to the rapid expansion of saturated areas and the increase in connectivity. Only after both the groundwater response and the development of saturated areas the tile drain system respond. This temporal sequence is also evident during the recession, where saturated areas contract and connectivity decreases before tile drain discharge reaches its peak.

### Connected and disconnected events

Analysis and visual inspection of camera images allow the identification of events which, during the events, have connected or disconnected saturation patterns in the downslope direction of the hillslope (Table 3). From the analysis of 12 runoff events in the period from January 2015 to December 2017, four events are classified as connected. An example of a connected saturation area pattern is presented on Fig. 5.

**Fig. 8:**
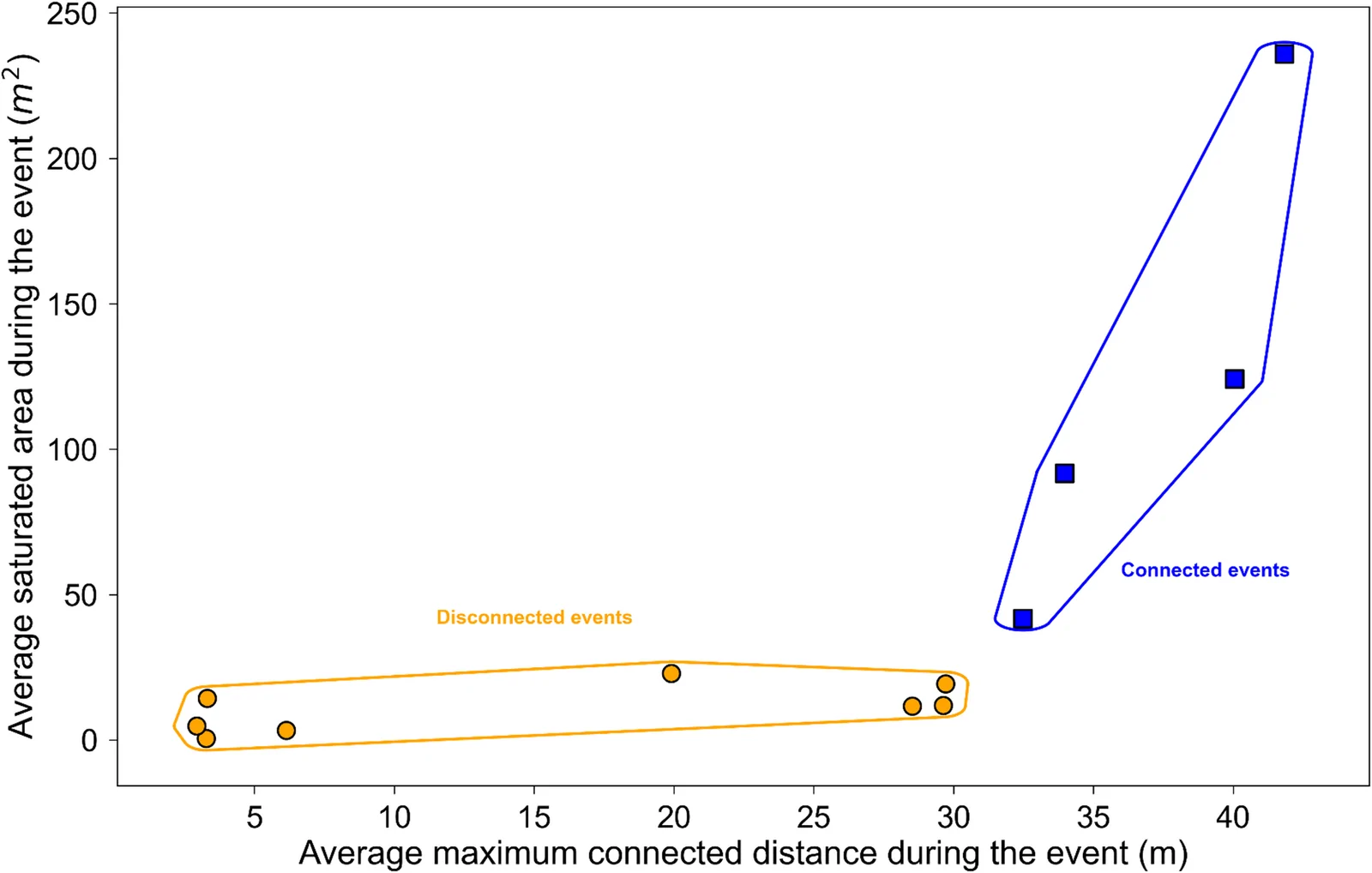
Grouping of events into connected and disconnected. The events in the orange outline are classified as disconnected, and the events in the blue outline are classified as connected.

Figure 8 compares the average saturated area and maximum connected distance—${h}_{max}$, during the events for both groups of events. The relationship shows that disconnected events have an average saturated area below 40 m^2^ and average ${h}_{max}$ is in the range between 2 and 30 m. Connected events have an average saturated area exceeding 50 m^2^ and the maximum connected distance during the events exceeds 32 m.

**Table 3:** Values for the observed characteristics during the events.

Event ID	Date	A_s_	I	Average A_s_	Average hmax	Connected
1	02/03/2015	238.4	8.46	91.66	33.97	Yes
2	02/04/2015	25.6	4.23	22.94	19.9	No
3	25/07/2016	6.6	4.06	11.71	28.52	No
4	28/07/2016	48.7	1.32	11.92	29.62	No
5	05/08/2016	28.8	1.92	14.42	3.31	No
6	10/08/2016	130.4	3.69	41.78	32.48	Yes
7	04/10/2016	7.4	3.09	3.35	6.13	No
8	20/10/2016	43.7	4.43	19.42	29.71	No
9	12/11/2016	206.9	8.87	124.2	40.05	Yes
10	25/12/2016	0.2	1.69	0.52	3.27	No
11	28/12/2016	99.82	6.58	4.9	2.93	Bo
12	21/02/2017	274.6	8.6	235.97	41.83	Yes

A_s_—Maximum saturated area corresponding to the peak of tile drain discharge (m^2^); I—Integral connectivity scale corresponding to the peak of tile drain discharge (m); Average A_s_—Average saturated area captured during the event (m^2^); Average hmax—Average maximum connected distance between two pixels during the whole event (m); Connected—Indicates whether there was observed connected overland flow downhill of the hillslope.

**Fig. 9:**
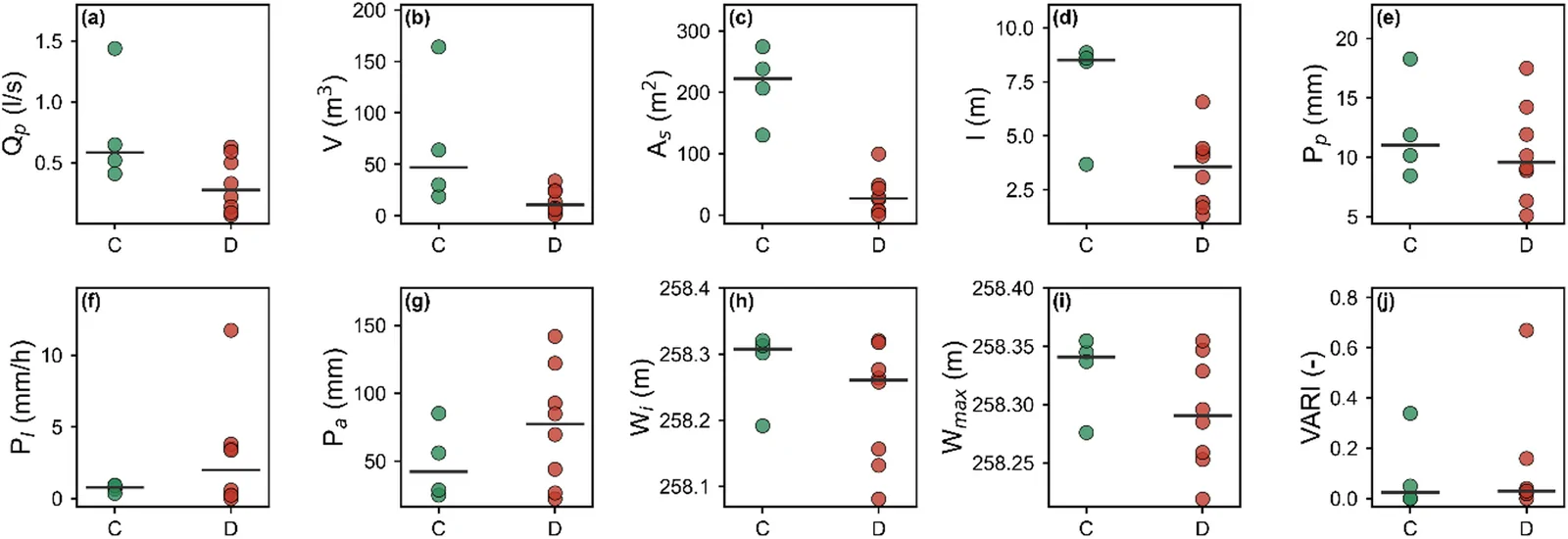
Overview of event characteristic distributions in regards to connected and disconnected events. The green dots represent the connected events, and the red dots represent the disconnected events. The figures show the event responses for: (**a**) Peak tile drain discharge, (**b**) Total volume drained, (**c**) Saturated area, (**d**) Integral connectivity scale, (**e**) Rainfall up to the peak (**f**) Average rainfall intensity, (**g**) 30-day antecedent rain, (**h**) Initial groundwater level, (**i**) Maximum groundwater level, (**j**) VARI index.

Figure 9 shows the distributions of the event characteristics for the connected and disconnected events. The connected events tend to have larger values for peak tile drain runoff, total volume drained, saturated area, and the integral connectivity scale (Fig. 9a–d), as well as initial and maximum event groundwater levels (Fig. 9h–i). The disconnected events have larger variability of rainfall characteristics (event rainfall, intensity, and antecedent rainfall), vegetation index as well as initial and maximum event groundwater levels.

### Factors controlling tile drain runoff dynamics

**Fig. 10:**
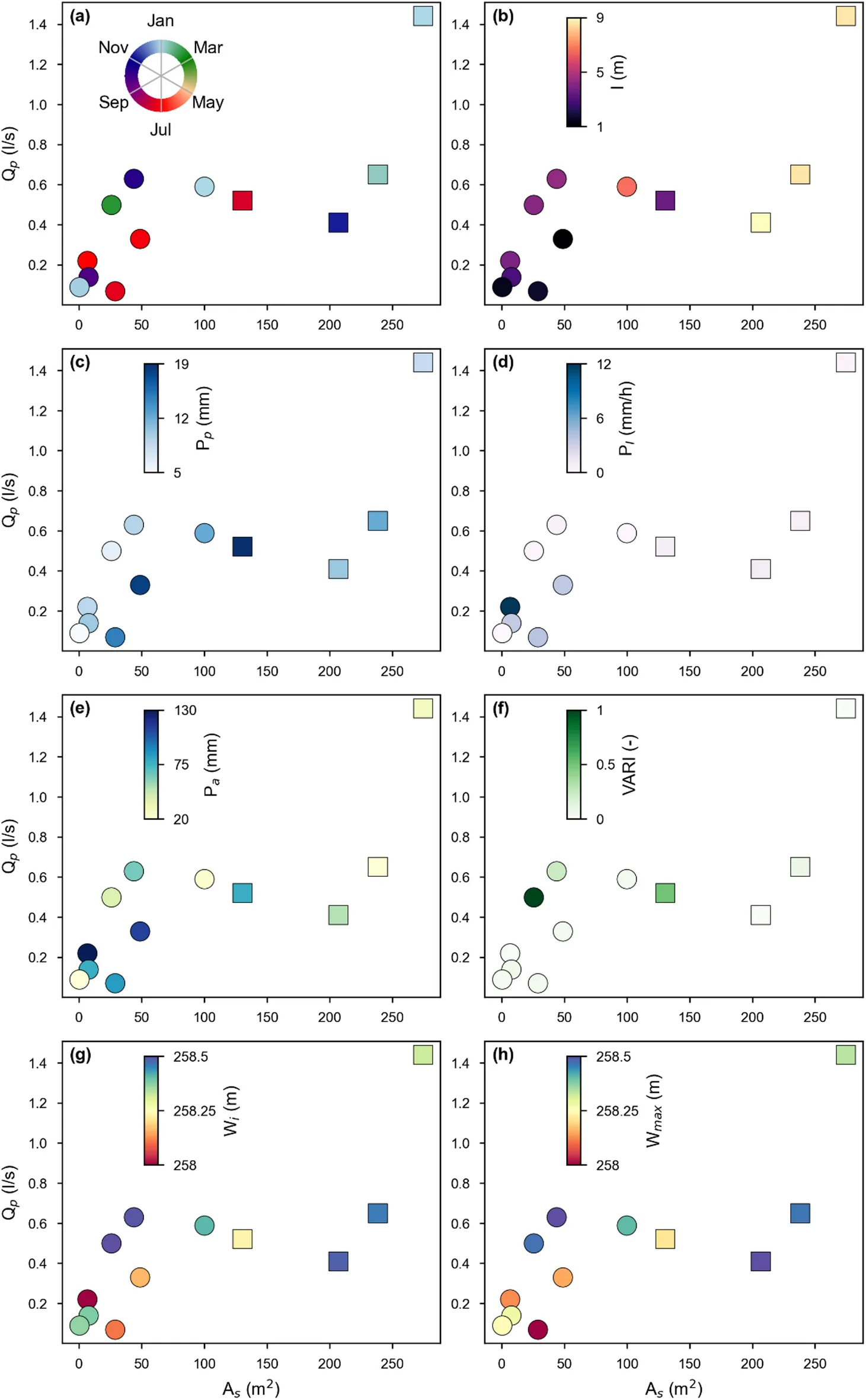
Peak tile drain discharge plotted against saturation area for the analyzed events. The events with the square marker are connected events, and event with the circle marker are disconnected. Colour maps show the distribution of different hydrological characteristics: (**a**) Seasonal distribution of identified events over the year. (**b**) Integral scale connectivity. (**c**) Rainfall up to the peak. (**d**) Average rainfall intensity. (**e**) 30-day antecedent rain. (**f**) Mean VARI index. (**g**) Initial groundwater levels. (**h**) Maximum groundwater levels.

**Fig. 11:**
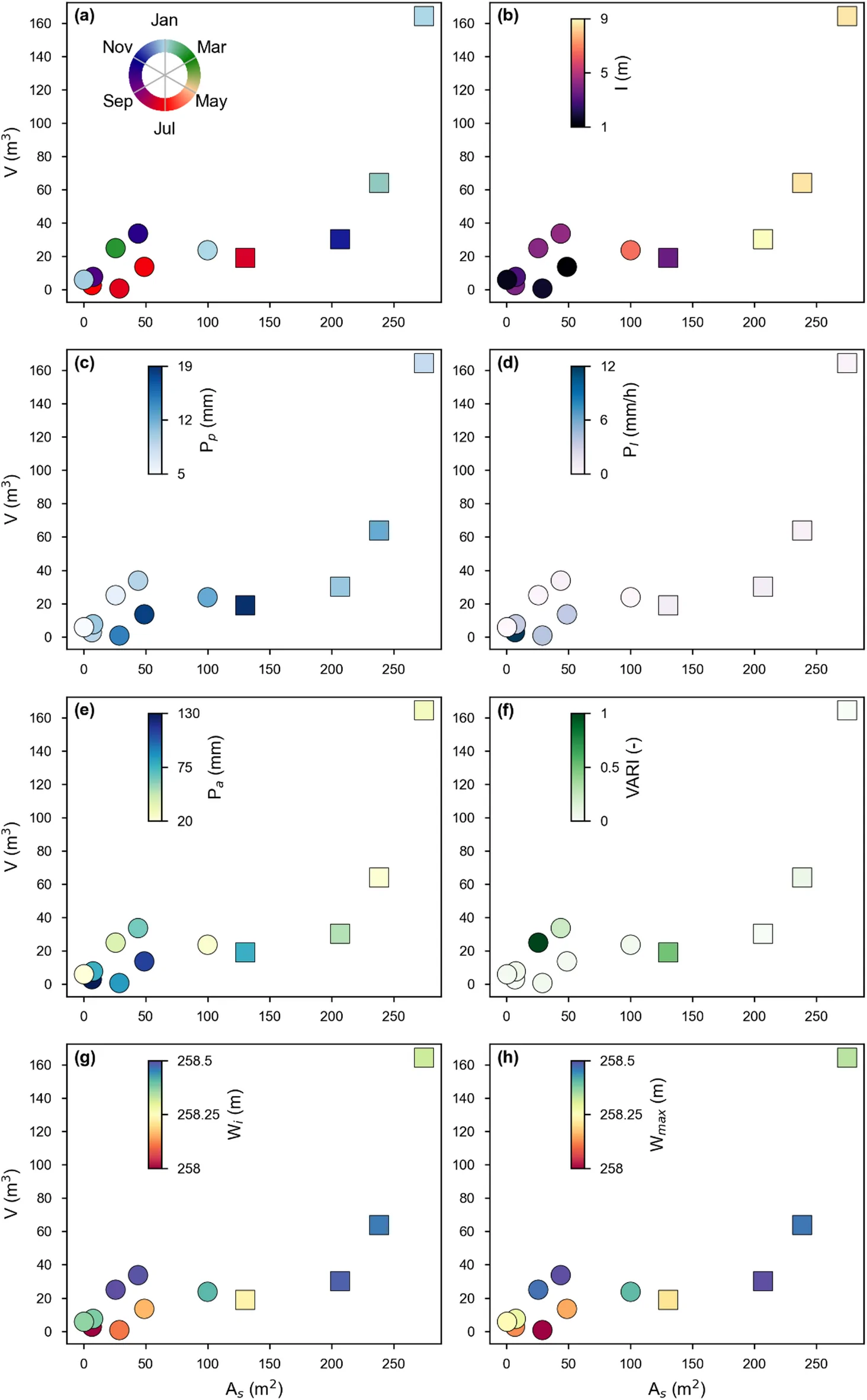
Event volume plotted against saturation area for the analyzed events. The events with the square marker are connected events, and event with the circle marker are disconnected. Colour maps show the distribution of different hydrological characteristics: (**a**) Seasonal distribution of identified events over the year. (**b**) Integral scale connectivity. (**c**) Rainfall up to the peak. (**d**) Average rainfall intensity. (**e**) 30-day antecedent rain. (f) Mean VARI index. (**g**) Initial groundwater levels. (**h**) Maximum groundwater levels.

**Fig. 12:**
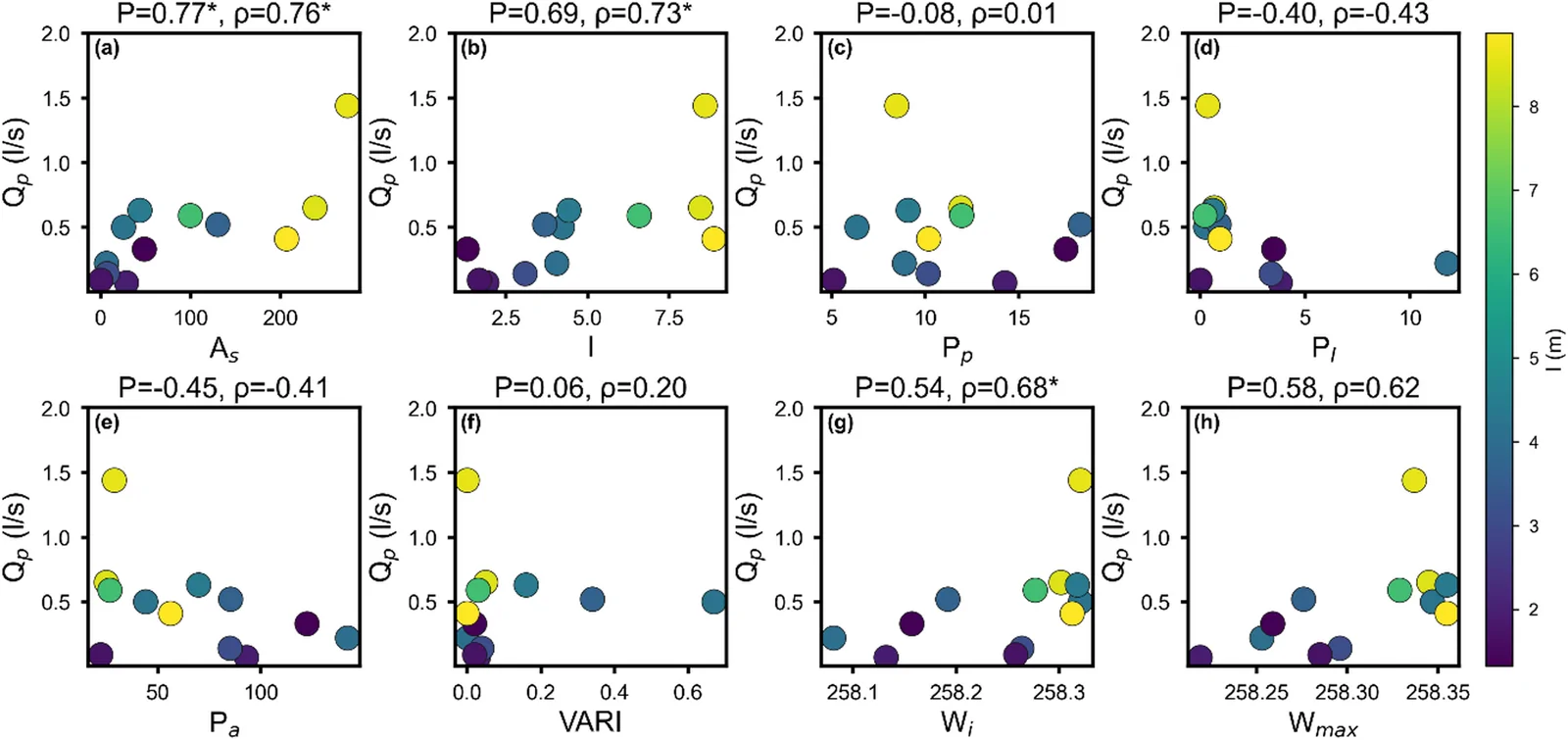
Correlations of peak tile drain discharge to the observed camera and hydrological variables: (**a**) Saturated area, (**b**) Integral connectivity scale, (**c**) Rainfall up to the peak, (**d**) Rainfall intensity, (**e**) 30-day antecedent rain, (**f**) VARI index, (**g**) Initial groundwater level, (**h**) Maximum groundwater level. The subplots are shown with integral scale colour mapping. P indicates the Pearson r, and *ρ* indicates the Spearman r, the stars next to the correlation values indicate the Benjamini-Hochberg corrected statistical significance, with one star indicating *p* < 0.05, and no stars in the case of *p* ≥ 0.05.

**Fig. 13:**
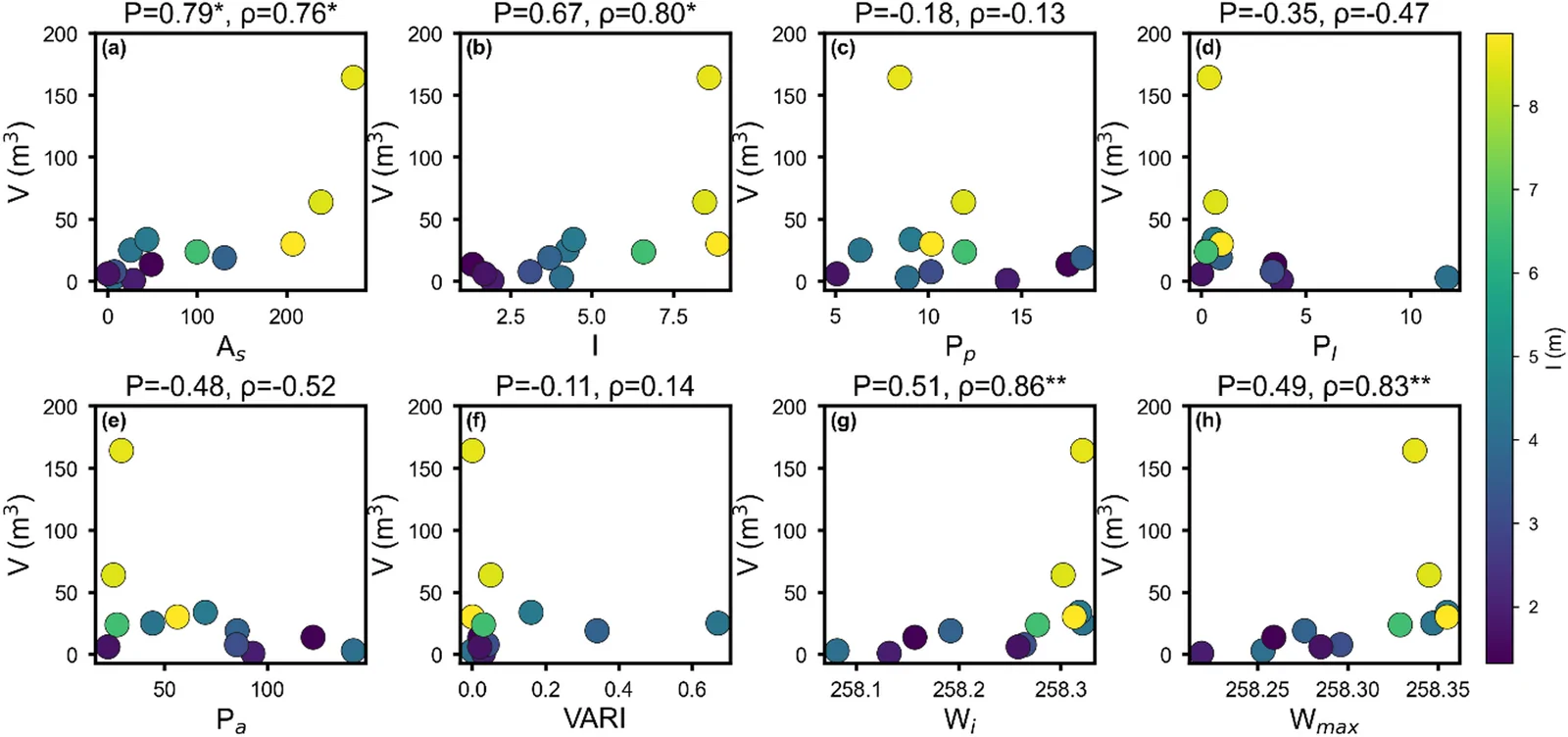
Correlations of event volume to the observed camera and hydrological variables: (**a**) Saturated area, (**b**) Integral connectivity scale, (**c**) Rainfall up to the peak, (**d**) Rainfall intensity, (**e**) 30-day antecedent rain, (**f**) VARI index, (**g**) Initial groundwater level, (**h**) Maximum groundwater level. The subplots are shown with integral scale colour mapping. P indicates the Pearson r, and *ρ* indicates the Spearman r the stars next to the correlation values indicate the Benjamini-Hochberg corrected statistical significance, with two stars indicating *p* < 0.01, one star indicating *p* < 0.05, and no stars in the case of *p* ≥ 0.05.

Figures 10 and 11 show the relationship between maximum event saturated area estimated from camera images and tile drain runoff peak (Fig. 10) and event volume (Fig. 11) for different event characteristics (i.e., colour of the symbols in the panels). Squares and circles indicate connected and disconnected events, respectively. The results indicate that, while the tile drain response increases with maximum event saturated area for all events, the response is more significant for the connected events. This particularly holds for event volume response (Fig. 11), where the disconnected event with the highest saturated area (99.82 m^2^) drained 23.8 m^3^, and the event with the highest saturated area (274.6 m^2^) drained 164.3 m^3^; a 2.75-fold increase in saturated area yielded a 6.9-fold increase in drained volume. This response increase is less pronounced for peak discharge (Fig. 10), with a 2.44-fold increase in peak discharge for the same saturated area increase. In terms of the explanatory power, the saturated areas explained 59% and 62% of the peak tile drain discharge and event volume behaviour, respectively (Figs. 12a and 13a). The explanatory power is understood to be the square of the Pearson coefficients. Notably, peak tile drain discharge has a consistently higher statistical power (with a mean of 0.88 to the observed variables) as compared to event volume (with a mean of 0.64 to the observed variables); meaning that peak tile drain discharge produces more clearly distinguishable relationships with the observed variables. The most likely cause of this discrepancy is the methodology used to determine the end of an event, which, in turn controls the total event volume; this, however, suggests that conclusions about event volume should therefore be interpreted with more caution than those for peak discharge. With the water infiltrating into the subsurface directly above the tile drains, the causes of the missing explanatory power are the preferential subsurface pathways^46^, as well as the macropores that occur in summer when the topsoil dries and cracks^43^. As the groundwater piezometers represent point-data they are not individually helpful for investigating subsurface pathways, however, installation of additional piezometers along the transect of the tile drain area is planned to help with understanding the infiltration processes.

Both tile drain discharge response metrics increase with rising integral scale connectivity (Figs. 10b and 11b), with 3 of the 4 connected events having a connectivity value over 8 m. Integral scale connectivity also explains 47% and 44% of the peak tile drain discharge and event volume behaviour, respectively (Figs. 12b and 13b). Most of the connected events have a larger connectivity scale as compared to the disconnected events, this is understood to also be a consequence of the relation of saturated area to the connectivity index, with a threshold of saturated area being surpassed and the sharp rise of the connectivity index. This suggests that the connection of saturated areas is playing a role in tile drain discharge response, and not just the gross saturated area that forms on the surface.

The results suggest that none of the rainfall characteristics, or the VARI index show any significant relation to the tile drain response (Figs. 10c–f, 11c–f, 12c–f and 13c–f). However, the threshold behaviour shown regarding the connectivity of the events (Fig. 8) is not present for the rainfall characteristics; with the connected and disconnected events occurring over the entire range of the characteristics. The event with the largest rainfall up to the peak is a connected event with the lowest saturated area, and the other connected events having values lower that some disconnected events. The event with the largest rainfall intensity is a disconnected event with very small saturated area and tile drain response, whereas the connected events all have a rainfall intensity below 1 mm/h. Similarly, the event with the largest value of antecedent rain is disconnected with a low saturated area and tile drain response. The distribution of antecedent rain for the connected events is interesting, with the connected event with the highest antecedent rain having the lowest saturated area, and the remaining connected events having lower antecedent rainfall values, which is a consequence of groundwater levels.

The high initial and maximum groundwater levels for the connected event indicate that the event groundwater levels are a strong predictor of tile drain discharge response (Figs. 10g, h and 11g, h) with the highest tile drain discharge responses occurring in high event groundwater levels; in contrast to the camera characteristics, their behaviour appears to be non-linear over all of the analyzed events (Figs. 12g, h and 13g, h). With 3 of the 4 saturated events occurring in high groundwater level conditions, of interest is Event #6, the connected event with the lowest saturated area (on Figs. 10 and 11). This event occurred in drier conditions, as indicated by the groundwater levels, and also registered the largest amount of rainfall up to the peak, despite having low rainfall intensity. Coupled with the not particularly large antecedent rain (Figs. 10e and 11e), the data suggests that this event occurred as a consequence of infiltration excess (Supplementary Figure S1 and Supplementary Figure S2).

To account for the multiple pairwise comparisons, p-values were adjusted using the Benjamini-Hochberg procedure at FDR = 0.05 within each correlation method. Under Spearman correlation, the relationships of saturated area and integral connectivity scale with both peak discharge and event volume remained significant after correction, as did those of initial and maximum groundwater levels with event volume. Under Pearson correlation, only the relationships of saturated area with both response metrics survived the correction, reflecting the greater sensitivity of the Pearson coefficient to the small sample size and to the influential observations identified above. None of the rainfall characteristics or the VARI index were significant under either method after BH correction. Fisher-z 95% confidence intervals (Supplementary Table S1) are wide for all correlations as a consequence of the limited number of events (*n* = 12), and should be interpreted accordingly; the intervals nonetheless confirm that the strongest associations - between saturated area, connectivity, and groundwater on one hand and tile drain response on the other - exclude zero under the rank-based analysis.

## Discussion and conclusion

This study explores the potential of saturated area observations in understanding tile drainage response to precipitation events. From the perspective of the temporal evolution of saturated areas, their relation to tile drain dynamics is twofold: Firstly, saturated areas will form on the hillslope prior to the activation of the tile drain during a rain event, as well as begin dissipation as the tile drain hydrograph starts receding (as shown on Fig. 7); secondly, a large jump in the time series of saturated areas is indicative of the change in the connectivity of the saturated areas, which leads to the water flowing from the hillslope, which results in larger tile drain responses, events exhibiting such behaviour have been labelled as “connected”.

To illustrate the importance of time-resolved saturated area dynamics and connectivity, a representative event was selected that was fully captured during daylight hours with clear visibility of saturated areas. This makes it well suited for examining the processes governing saturated area generation. All events analysed in this study exhibit similar dynamics, with the primary sources of variability being antecedent conditions and total event rainfall. Because saturated area extent can change on timescales of minutes, continuous monitoring is essential. Time-lapse camera observations offer a low-cost, low-maintenance approach to hillslope monitoring and are well suited for automation^52^, enabling continuous and near real-time processing of imagery. This near real-time processing offers an advantage to the traditional methods because it offers spatial information in a high temporal resolution, the spatial information provided is directly contrasted against the point measurements of piezometers; in addition, piezometers provide only information about the subsurface, whereas camera observations of the surface potentially present a link between the surface and the subsurface. Integrating a pre-screening step that would enable automatic detection of overly vegetated areas is possible through calculating the VARI index for every timestep, while for snow cover, the methodology developed by^53^ could be integrated into the raw camera observations to identify events where the snow cover would provide false positives for saturated area observations. Furthermore, in future research we will be testing potential deep learning methods for identification of saturated areas and possible pre-screening for application of the method used in this study. Two main limitations arise when applying this method. First, snow cover obscures saturated areas, complicating the interpretation of tile drain responses. Following snowfall, snow may persist before rapidly infiltrating during warming, leading to outlier behaviour relative to events without snow. Second, the approach assumes that all saturated areas are visible. This restricts the number of usable events, as tile drain discharge may occur without surface saturation in more permeable soils. Conversely, on clay soils, infiltration excess runoff may occur without a tile drain response^54^. To mitigate this limitation, the VARI index was used to exclude events during periods when vegetation height prevented reliable observations of saturated areas. An additional limitation of the method is found in the lack of water source identification^55^. Using a thermal imaging method identifies water emerging from the riparian zone and mixing with the cold water from the melting snow cover, such information would help delineate the main process(es) driving saturated area generation. The area covered by the entire tile-drain system that drains into the Frau1 flume, with a drainage area of 3.1 ha^56^, shares similar topographic characteristics^46^, with the entire camera view covering 20.5% of the tile-drain system, and this study focusing on a 0.3 ha, or 9.6% of the total area; this area is therefore assumed to be representative of the entire tile-drained area. The extrapolation of this method to the remaining tile-drain system is planned for future studies by including camera observations of the other tile-drained areas.

The results presented on Figs. 10 and 11 show that drained event volume increases more with the events becoming connected than the peak tile drain discharges. This is a consequence of the physical behaviour of the connected events, which begin flowing down the hillslope, at which point water from the thalweg accumulates and dissipates over the drainage area, as the tile drainages are not located only directly under the thalweg (Fig. 1). This dissipation of the flowing water over a larger area allows the water to infiltrate quicker as the area of the infiltration front expands and is faster to be collected by the tile drainages, which is reflected in a clear increase in the tile drain discharge response characteristics. An additional consideration is the separation of events into connected and disconnected. The results indicate that the best metrics for delineating event connection are the average maximum connected distance, and the average saturated area during the event; with the values used for the connection delineation in this study, of 32 m and 50 m^2^, respectively, being a consequence of the microtopography of the observed area. A microtopographical consideration in agricultural fields is rill channelization (for example through tractor tracks) which can lead to saturated areas forming and then not changing for a prolonged period of time as the deep rills are able to channel and hold a larger amount of water, thus interfering with the results of the saturated area—tile drain discharge characteristics relations.

The groundwater levels show the best predictive performance out of the hydrological variables, with them having a spearman *ρ* > 0.6 and > 0.8 for peak tile drain discharge and drained event volume, respectively, with a lower Pearson r, suggesting a larger degree of non-linearity in tile drain response behaviour to groundwater levels. This, in turn, suggests that the main driver of saturated area generation is saturation excess. However, as discussed previously, Event #6 appears to come about due to infiltration excess (Supplementary Figure S1 and Supplementary Figure S2), which shows that while saturation excess is the most common driver of saturated area and tile drain response generation, in particular initial condition cases infiltration excess can also lead to connected events. Because the limited number of available events means the statistical analyses are more sensitive to extreme values, it would be interesting to expand the available dataset in the future, once more observations become available. The contrast between the Pearson and Spearman coefficients in the present dataset, together with the comparatively broader survival of Spearman correlations under the Benjamini-Hochberg correction, indicates that several of the linear relationships are partly driven by individual influential events, while the underlying monotonic associations are more robust. This is one reason for reporting both coefficients alongside one another on Figs. 12 and 13.

This study finds that the observed saturated areas, and the integral connectivity scale are tile drain response predictors on par with the groundwater levels, with both of them being significantly better than the rainfall characteristics. These findings are in agreement with^37^, who found that antecedent groundwater conditions to be more influential than rainfall characteristics. Notable is that the observed saturated areas and the integral connectivity scale showed a more linear relation to the tile drain response characteristics (with the exception of the integral scale to drained volume), whereas the groundwater levels suggest a prominent non-linear tile drain response to increasing groundwater levels.

Previous studies examining the role of soil moisture generally report a positive non-linear relationship with peak tile drain discharge^10,39–41^. Using 30-day antecedent rain as a proxy for soil moisture, this study found no significant correlation, linear or non-linear, between 30-day antecedent rain and the analysed events. A large body of literature has focused on tile drain responses to both incidental and antecedent rainfall characteristics^16,37–41^. The findings of this study align with^39^, who reported more continuous behaviour in rainfall-tile drain responses, with tile drain discharge occurring across the full range of rainfall conditions. The absence of a significant relationship between average rainfall intensity and tile drain discharge is consistent with^37^ and^57^, but contrasts with^41^, who observed increasing tile drain discharge with increasing rainfall intensity. Unlike several previous studies^16,37,39^, no significant positive correlation is found between bulk rainfall and tile drain discharge.

There exist several possible research directions: (i) Moving away from natural lighting conditions into more controlled lighting conditions, or even into the infrared spectrum. (ii) Improvement of the camera quality, allowing for a larger or more detailed observation area. (iii) Usage of different connectivity indices, representing different physical characteristics. (iv) Application of saturated area observation methodology to surface runoff predictions. (v) Integrating multi-directional camera observations, for understanding the movement of water in the larger system.

## Supplementary Information

Below is the link to the electronic supplementary material.

Supplementary Material 1.

## Data Availability

The data supporting the findings of this study is available on request from the corresponding author.
