# Supplementary material for: Understanding tile drain discharge dynamics through saturated area observation
Source: Sci Rep. 2026 Jun 11;16:26720. doi: 10.1038/s41598-026-56451-3 (PMC13507095; doi:10.1038/s41598-026-56451-3)
Supplement: Supplementary file 1 [file 41598_2026_56451_MOESM1_ESM.docx]

Supplementary Table S1 Correlation statistics between tile drain discharge response variables and explanatory variables. Tile drain response (TDR) - Tile drain discharge metric used as the response variable in the correlation analysis; Explanatory variable (EV) - Variable tested against the tile drain response; Correlation method - Correlation approach used in the analysis; Correlation coefficient (r) - Strength and direction of the relationship between the two variables; Raw p-value (p) - Unadjusted significance level associated with the correlation test; CI lower - Lower bound of the 95% confidence interval for the correlation coefficient estimated using Fisher’s z transformation; CI upper - Upper bound of the 95% confidence interval for the correlation coefficient estimated using Fisher’s z transformation; BH-adjusted p-value (within method) - Benjamini-Hochberg adjusted p-value calculated separately for each correlation method; BH-adjusted p-value (overall) - Benjamini-Hochberg adjusted p-value calculated across all performed tests.

| **TDR** | **EV** | **Correlation method** | **r** | **p** | **CI lower** | **CI upper** | **BH-adjusted p-value (within method)** | **BH-adjusted p-value (overall)** |
| --- | --- | --- | --- | --- | --- | --- | --- | --- |
| Q_P_ | A_S_ | pearson | 0.765 | 0.004 | 0.341 | 0.930 | 0.030 | 0.024 |
| Q_P_ | I | pearson | 0.692 | 0.013 | 0.196 | 0.906 | 0.066 | 0.043 |
| Q_P_ | W_max_ | pearson | -0.083 | 0.798 | -0.627 | 0.516 | 0.851 | 0.851 |
| Q_P_ | W_i_ | pearson | -0.396 | 0.202 | -0.791 | 0.230 | 0.294 | 0.281 |
| Q_P_ | P_A_ | pearson | -0.454 | 0.138 | -0.815 | 0.162 | 0.222 | 0.222 |
| Q_P_ | P_I_ | pearson | 0.537 | 0.072 | -0.053 | 0.849 | 0.191 | 0.164 |
| Q_P_ | P_P_ | pearson | 0.576 | 0.050 | 0.003 | 0.864 | 0.160 | 0.123 |
| Q_P_ | VARI | pearson | 0.055 | 0.864 | -0.536 | 0.610 | 0.864 | 0.892 |
| V | A_S_ | pearson | 0.789 | 0.002 | 0.393 | 0.938 | 0.030 | 0.024 |
| V | I | pearson | 0.673 | 0.016 | 0.162 | 0.900 | 0.066 | 0.048 |
| V | W_i_ | pearson | -0.181 | 0.572 | -0.684 | 0.438 | 0.705 | 0.705 |
| V | W_max_ | pearson | -0.348 | 0.268 | -0.768 | 0.283 | 0.358 | 0.358 |
| V | P_A_ | pearson | -0.476 | 0.118 | -0.825 | 0.135 | 0.210 | 0.210 |
| V | P_I_ | pearson | 0.504 | 0.094 | -0.098 | 0.836 | 0.210 | 0.189 |
| V | P_P_ | pearson | 0.485 | 0.110 | -0.123 | 0.828 | 0.210 | 0.207 |
| V | VARI | pearson | -0.111 | 0.731 | -0.644 | 0.494 | 0.836 | 0.807 |
| Q_P_ | A_S_ | spearman | 0.762 | 0.006 | 0.318 | 0.932 | 0.019 | 0.027 |
| Q_P_ | I | spearman | 0.734 | 0.009 | 0.259 | 0.923 | 0.024 | 0.036 |
| Q_P_ | W_i_ | spearman | 0.007 | 0.991 | -0.582 | 0.591 | 0.991 | 0.991 |
| Q_P_ | W_max_ | spearman | -0.434 | 0.161 | -0.813 | 0.205 | 0.235 | 0.246 |
| Q_P_ | P_I_ | spearman | -0.406 | 0.193 | -0.802 | 0.238 | 0.257 | 0.280 |
| Q_P_ | P_A_ | spearman | 0.688 | 0.013 | 0.170 | 0.908 | 0.031 | 0.043 |
| Q_P_ | VARI | spearman | 0.639 | 0.025 | 0.083 | 0.891 | 0.051 | 0.068 |
| Q_P_ | P_P_ | spearman | 0.201 | 0.530 | -0.437 | 0.705 | 0.653 | 0.679 |
| V | W_i_ | spearman | 0.762 | 0.006 | 0.318 | 0.932 | 0.019 | 0.027 |
| V | W_max_ | spearman | 0.797 | 0.003 | 0.395 | 0.943 | 0.017 | 0.024 |
| V | I | spearman | -0.133 | 0.683 | -0.668 | 0.492 | 0.729 | 0.781 |
| V | A_S_ | spearman | -0.469 | 0.127 | -0.828 | 0.163 | 0.204 | 0.215 |
| V | P_A_ | spearman | -0.517 | 0.089 | -0.847 | 0.099 | 0.158 | 0.189 |
| V | P_I_ | spearman | 0.864 | 0.000 | 0.563 | 0.963 | 0.005 | 0.009 |
| V | VARI | spearman | 0.842 | 0.001 | 0.505 | 0.956 | 0.005 | 0.009 |
| V | P_P_ | spearman | 0.141 | 0.661 | -0.486 | 0.672 | 0.729 | 0.781 |

Supplementary Figure S1 Soil moisture data for profile #5 over the course of Event #6

Supplementary Figure S2 Soil moisture data for profile #16 over the course of Event #6
